# Low Elastin Content of Carotid Plaques Is Associated with Increased Risk of Ipsilateral Stroke

**DOI:** 10.1371/journal.pone.0121086

**Published:** 2015-03-24

**Authors:** Giuseppe Asciutto, Nuno V Dias, Andreas Edsfeldt, Mihaela Nitulescu, Ana Persson, Marie Nilsson, Pontus Dunér, Jan Nilsson, Isabel Gonçalves

**Affiliations:** 1 Vascular Centre Malmö-Lund, Skåne University Hospital, Malmö, Sweden; 2 Experimental Cardiovascular Research Unit, Clinical Research Centre, Dept. of Clinical Sciences Malmö, Lund University, Malmö, Sweden; 3 Department of Cardiology, Skåne University Hospital, Malmö, Sweden; Brigham and Women's Hospital, Harvard Medical School, UNITED STATES

## Abstract

**Objectives:**

Atherosclerotic plaques with a low content of connective tissue proteins are believed to have an increased risk of rupture and to give rise to clinical events. The aim of the present study was to investigate if the content of elastin, collagen and of the matrix metalloproteinase (MMP) −1, −3, −9 and −12 in plaques removed at surgery can be associated with the occurrence of ipsilateral symptoms.

**Methods:**

The atherosclerotic plaques of 221 patients undergoing carotid endarterectomy were analyzed and their composition was related to the incidence of preoperative, intraoperative and postoperative neurological events.

**Results:**

Elastin, collagen and MMP-12 contents were lower in males and diabetic patients. Elastin (P .010), MMP-3 (P .008) and MMP-9 (P < .0001) were lower, while MMP-1 (P .004) and MMP-9 (P .002) were higher in plaques of patients with preoperative symptoms, even after correction for the time between the occurrence of symptoms and surgery. Elastin and MMP-12 decreased (r = −0.17, P .009 and r = −.288, P <.0001 respectively) while MMP-1 (r = 0.17, P .012) and MMP-9 (r = .21 P <.0001) increased with age. After a mean follow-up time of 39.6 ± 16.6 months, 7.7% of patients had suffered one or multiple ipsilateral neurological events. Patients with plaque elastin levels lower than the median (52 mg/g) had increased post-operative incidence of ipsilateral stroke (P for trend 0.009 using Log Rank Chi-square test). This finding was confirmed when controlling for age, gender, hypertension, diabetes, smoking, pre-operative symptoms and statin usage in a Cox Proportional Hazard model (hazard ratio 7.38, 95% C.I. 1.50–36.31).

**Conclusions:**

These observations support the concept that elastin may be important for plaque stability, and suggest that a low plaque content of elastin is associated with a higher risk for ipsilateral stroke.

## Introduction

Several randomized trials [1–3] have concluded that carotid endarterectomy (CEA) is effective in reducing the risk of stroke and death in patients with severe symptomatic high-grade carotid artery stenosis. The stability of atherosclerotic plaques and the related neurological symptoms are determined by a complex interplay among inflammation, extracellular matrix (ECM) degradation by proteases such as matrix metalloproteinase (MMP), cell death and synthesis of new connective tissue proteins. [4] The ECM proteins elastin and collagen are of particular importance for maintaining plaque stability. Elastin is organized into fibers contributing to the elastic properties of the arterial wall, while collagen is its main load-bearing component. The elastic fibers undergo degradation and fragmentation with age and disease, with parallel collagen accumulation, leading to increased stiffness of the arterial wall, [5] which is an important, independent predictor of cardiovascular (CV) mortality in patients with hypertension, as well as end-stage renal failure and diabetes. [6]

An unstable plaque phenotype can, in theory, result in the occurrence of intraoperative symptoms as caused through mechanical stress by vessel manipulation during CEA. Furthermore, the extension of the atherosclerotic lesions in diseased arteries is beyond the carotid bifurcation. Therefore, we theorized that reduced plaque levels of the components of the ECM could be associated with an increased risk of related events.

In order to prove this hypothesis, the elastin, collagen and MMPs content of human carotid plaques harvested during CEA was analyzed and correlated with the incidence of early and late neurological events, taking account of comorbid conditions and the patients´ neurologic presentation.

## Methods

Two hundred-twenty-one patients aged 69.7 ± 8.4 years who underwent CEA between October 2005 and October 2009 at our Vascular Department were included in this study. Written informed consent was given by each patient, and the study was approved by the local ethical committee (Decision numbers 472/2005 and 16209/2012).

The degree of carotid artery stenosis was preoperatively assessed with ultrasound based on flow velocities as previously validated. [7] Indications for CEA as well as our routine medical treatment have been previously described. [8] Patients with ipsilateral carotid artery occlusion, radiation-induced stenosis, or restenosis after previous CEA or endovascular treatment were excluded. All patients were clinically assessed by an independent accredited neurologist preoperatively as well as at 30 days postoperatively, and upon the development of symptoms during follow-up. Patients were considered to have asymptomatic disease if they had no *amaurosis fugax* (AF), transient ischemic attacks (TIAs) or stroke in the six months prior to surgery.

Information about comorbidities and past medical history was obtained through standardized preoperative interviews and review of the medical records. Comorbidity factors such as hypertension, diabetes, peripheral artery disease (PAD), and smoking, as well as the body mass index (BMI), serum levels of triglycerides (TG), low-density lipoproteins (LDL) and high-density lipoproteins (HDL) were recorded. Intraoperative and perioperative events were analyzed from reviewing medical charts. The Swedish national inpatient health register was analyzed in order to identify postoperative neurologic events, with corresponding International Classification of Diseases, Tenth Revision (ICD-10) codes G45 and G46 from October 2005 to December 2010. This is a nationwide validated register in which more than 99% of all somatic (including surgery) and psychiatric hospital discharges are registered. [9] In doubtful cases, information was gained through telephone interviews and medical chart reviews. All causes of death were confirmed through the National Population Register.

### Definition of outcomes

All neurological ipsilateral events (i.e. fatal and non-fatal AF, TIA and stroke) were identified as described above and registered and analyzed singularly. Events occurring in the first 24 hours postoperatively were considered as procedure-related and assumed as intraoperative for analysis.

Patients suffering more than one episode of the same event (for example, patients with multiple strokes) were classified as suffering multiple events. In these cases only the first chronological event was taken into account in the survival analysis.

### Plaque sample preparation

The plaques were processed as described previously. [10–11] Briefly, plaques were removed by endarterectomy and immediately snap-frozen in liquid nitrogen. One-millimeter-thick fragments from the most stenotic region of the frozen plaques were removed for histologic analysis. The remaining parts of the plaque were weighed, cut into pieces while still frozen, and homogenized in 5 mL of a buffer consisting of 50 mmol/L Tris-HCl (pH 7.5), 0.25 mol/L sucrose, 2 mmol/L tris(2-carboxyethyl)phosphine HCl, 50 mmol/L NaF, 1 mmol/L Na-orthovanadate, 10 mmol/L Na-glycerophosphate, 5 mmol/L Na-pyrophosphate, protease inhibitor cocktail (Roche Complete, EDTA-free,), 1 mmol/L benzamidine, and 10 mmol/L phenylmethylsulfonyl fluoride.

### ECM components assessment in plaque homogenate

To measure elastin, a Fastin Elastin assay was used (Biocolor, Carrickfergus, Northern Ireland, UK). Plaque homogenate (30μl) was mixed with cooled Elastin Precipitating Reagent (1000μl), incubated and centrifuged (10000g, 10 min). Supernatants were discarded, and Fastin Dye Reagent and ammonium sulfate (1000 μl+100μl) were added. The samples were mixed, centrifuged (12000g, 10 min), supernatants were discarded and Fastin Dissociation Reagent (1000μl) was added. The samples were measured on a Tecan Elisa plate reader (492 nm).

When measuring collagen, Sircol soluble collagen assay was used (Biocolor, Carrickfergus, Northern Ireland, UK). Samples (5μl) were added to 995 μl of Sircol Dye reagent, mixed, incubated (30 min) and then centrifuged (10000g, 10 min). This assay detects acid-soluble and pepsin-soluble collagens types I to V. The supernatants were discarded and alkali reagent (1000μl) was added. The samples were measured (absorbance 540nm) on a Tecan Elisa plate reader.

MMPs −1, −3 and −9) were analyzed in homogenate supernatants (25 μl) using Mesoscale human MMP ultrasensitive kit (Mesoscale, Gaithersbur, MD, USA), according to the manufacture´s instructions.

MMP-12 levels were analyzed by the Proximity Extension Assay (PEA) technique using the Proseek Multiplex CVD96x96 reagents kit (Olink Bioscience, Uppsala, Sweden) as previously described. [12] Data analysis was performed by a preprocessing normalization procedure using Olink Wizard for GenEx (Multid Analyses, Sweden). MMP-12 levels are presented as arbitrary units.

### Statistical analysis

Results were normalized to the wet weight of the plaque. Continuous variables are presented as mean ± standard deviation (SD) when not stated otherwise, while categorical variables are presented as percentages. Pearson´s Chi-square test was used for categorical variables. Student’s t-test and Pearson´s correlation were used for continuous variables whenever normally distributed, while a Mann-Whitney U test and Spearman´s rank correlation were used for non-normally distributed variables. Elastin and MMPs values were revealed to be non-normally distributed while collagen values were normally distributed. Simple and multiple linear regressions were used to explore the relationship between two or more variables. Freedom from postoperative events was calculated by life-tables according to Kaplan-Meier survival analysis. Correction for age, gender, hypertension, diabetes, smoking, the occurrence of pre-operative symptoms and statin usage was carried out through Cox regression analysis. A P-value of < .050 was considered statistically significant. Statistical analysis was performed using SPSS 20.0 (IBM Corp., Amonk, NY, USA).

## Results

Two hundred-twenty-one plaques removed by CEA were analyzed for total elastin and collagen content. Of these, two hundred-nine plaques were analyzed for MMP-1, 3- and 9 while only two hundred-one plaques for MMP-12 contents, due to lack of specimen material. Fifty-five % (n = 121) of the plaques were associated with preoperative symptoms, and among those, 7% (n = 15) had more than one preoperative neurological event. There were no differences in risk factor distribution between patients with and without preoperative symptoms.

**Fig 1:**
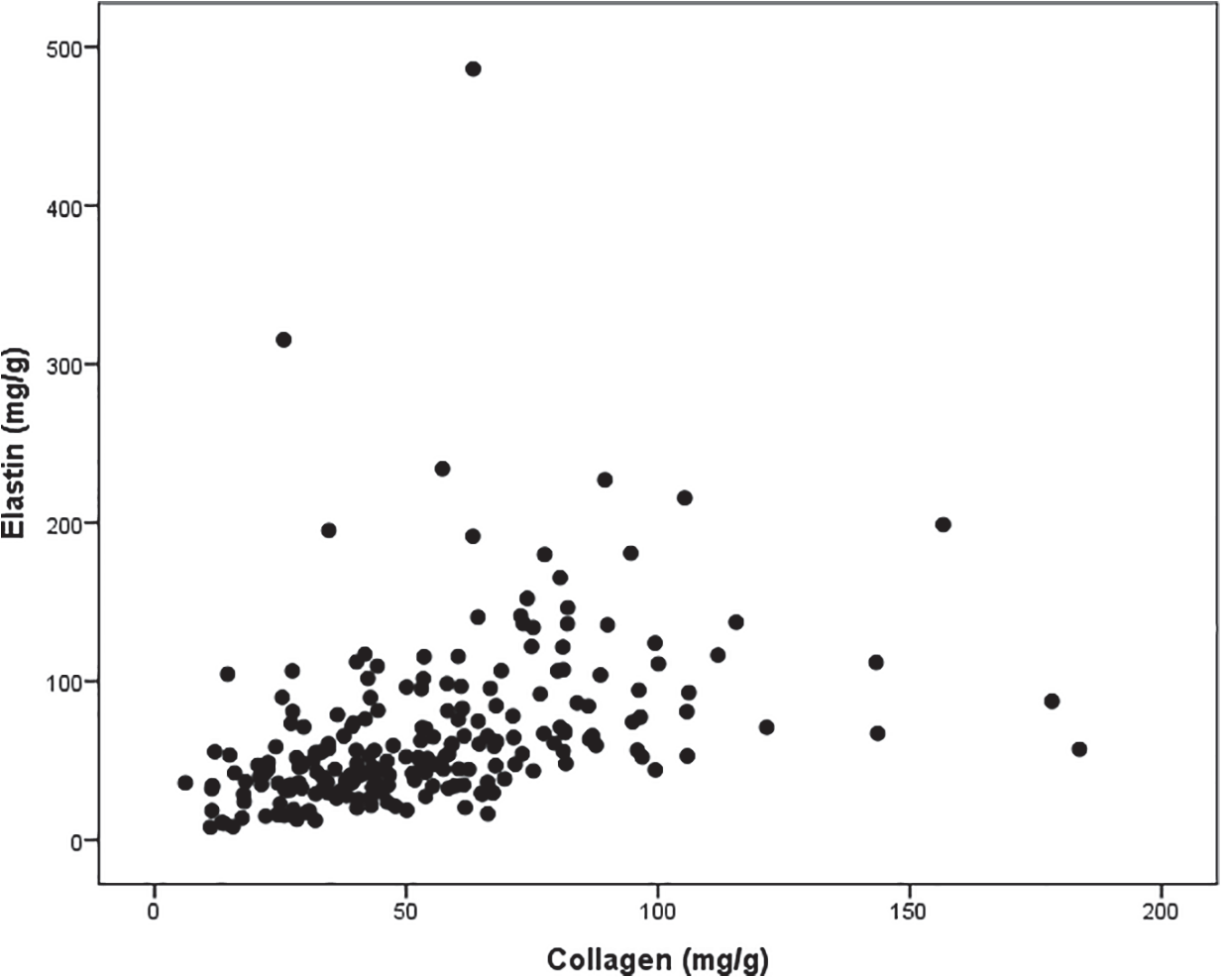
Scatter plot showing the positive correlation between elastin and collagen content (Spearman correlation r = .554, P <.0001).

There was a positive correlation between the plaque contents of elastin and collagen (r = .554, P < .0001; Fig. 1). MMP-9 plaque content correlated positively with MMP-1 (r = .599, P <.0001) and MMP-3 (r = .327, P <.0001) content and correlated inversely with MMP-12 content (r = −.362, P < .0001). MMP-3 levels correlated positively with elastin (r = .138, P .046), collagen (r = .260, P .0001) and MMP-12 (r = .176, P .015) content. MMP-12 levels correlated positively with elastin (r = .507, P <.0001) and collagen (r = .323, P <.0001) and inversely with MMP-1 (r = −.156, P .031) content.

The plaque contents of elastin, collagen and MMP-12 were lower while MMP-9 levels were higher in males than in females (Tables 1 and 2).

**Table 1:** Collagen and elastin content (mean ± standard deviation) expressed in mg/g analyzed for risk factors.

	Collagen	P	Elastin	P
Gender				
*Female*	60.7 ± 29.5	.026	83.4 ± 66.9	.00002
*Male*	51.4 ± 28.8		58.9 ± 43.5	
Hypertension				
*Yes*	54.5 ± 30.6	.975	66.6 ± 45.7	.249
*No*	54.4 ± 25.6		68.9± 73.3	
Diabetes				
*Yes*	44.6 ± 19.8	.00002	55.9 ± 35.3	.030
*No*	59.8 ± 32.2		72.9 ± 60.4	
Smoking				
*Current*	54.7 ± 24.2		71.3 ± 48.9	
*No*	54.9 ± 30.6	.951	70.9 ± 54.9	.386
*ex*	54.1 ± 31.9		62.5 ± 55.9	
Coronary artery disease ^a^				
*Yes*	52.1 ± 28.3	.327	61.3 ± 41.9	.243
*No*	56 ± 30		70.5 ± 59.5	
Lipid-lowering treatment				
*Yes*	55.5 ± 29.6	.165	66.1 ± 46.1	.499
*No*	47.4 ± 26.7		72.7 ± 89.1	
Preoperative symptoms				
*Yes*	52.5 ± 30.1	.248	58.2 ± 37.4	.010
*No*	57.1 ± 28.2		78.4 ± 67.6	

^a^ = stable and/or unstable angina, history of myocardial infarction and/or coronary artery surgery/angioplasty.

**Table 2:** MMPs content (mean ± standard deviation) analyzed for risk factors. expressed in ng/g for MMP-1, 3 and 9 and in arbitrary units/g for MMP-12.

	MMP-1	P	MMP-3	P	MMP-9	P	MMP-12	P
Gender								
*Female*	286.67 ± 435.71	.268	15.69 ± 20.5	.811	949.32 ± 153.32	.044	33985.03 ± 25353.66	.002
*Male*	196.38 ± 282.06		13.9 ± 13.9		955.23 ± 126.10		25776.62 ± 23741.09	
Hypertension								
*Yes*	206.43 ± 311.98	.051	14.36 ± 16.75	.624	910.25 ± 1252.25	.360	27300.27 ± 22916.45	.911
*No*	287.87 ± 416.34		14.93 ± 15.24		1099.63 ± 1626.22		31000.22 ± 28670.74	
Diabetes								
*Yes*	243.85 ± 387.41	.841	11.52 ± 10.28	.227	896.83 ± 1399.38	.636	22619.48 ± 18766.19	.011
*No*	215.84 ± 314.31		16.1 ± 18.66		984.27 ± 1329.39		31406.78 ± 26593.42	
Smoking								
*Current*	150.65 ± 182.08	.074	12.04 ± 11.87	.394	626.05 ± 722.06	.032	32089.78 ± 26277.02	.244
*No*	340.22 ± 463.23		17.18 ± 22.85		1276.99 ± 1271.4		24242.03 ± 22080.58	
*ex*	226.49 ± 350.32		14.92 ± 15.5		1028.58 ± 1630.62		27566.66 ± 24087.42	
Coronary artery disease ^a^								
*Yes*	170.1 ± 265.03	.023	14.28 ± 14.58	.965	780.03 ± 1363.85	.143	29360.17 ± 24672.88	.609
*No*	260.52 ± 378.48		14.68 ± 17.34		1062.18 ± 1343.44		27838.18 ± 24568.63	
(continue)Lipid-lowering treatment								
*Yes*	214.4 ± 328.69	.365	14.67 ± 16.41	.760	920.57 ± 1358.94	.133	28274.43 ± 23203.19	.218
*No*	299.16 ± 413.25		13.26 ± 15.97		1164.98 ± 1309.15		29169.79 ± 32492.09	
Preoperative symptoms								
*Yes*	276.85 ± 379.93	.004	12.29 ± 15.58	.008	1131.61 ± 1590.69	.002	23004.17 ± 21782.55	<.0001
*No*	158.21 ± 269.77		17.37 ± 16.9		717.56 ± 904.26		34521.21 ± 26060.07	

^a^ = stable and/or unstable angina, history of myocardial infarction and/or coronary artery surgery/angioplasty.

Plaques of patients who have experienced preoperative symptoms were characterized by lower elastin, MMP-3 and MMP-12 content as well as by higher levels of MMP-1 and MMP-9 when compared to those of asymptomatic patients. The elastin (r = −.179, P = .009) and MMP-12 (r = −.288 P <.0001) content of the plaques decreased while the MMP-1 (r = .174, P .012) and MMP-9 (r = .299, P<.0001) content increased with age. Significantly lower levels of elastin, collagen and MMP-12 were found in patients with diabetes as compared with non- diabetics. Current smokers have lower levels of MMP-9 compared to no- or past-smokers. Furthermore, when analyzing time interval between symptoms and CEA (22.2 ± 28 days), plaque content of collagen was significantly lower if the surgical treatment was performed in the first month after symptom onset (P = .007). There were no similar association for the elastin and MMPs content.

### Matrix proteins and incidence of neurological events

Two patients (0.9%) suffered an intraoperative (i.e. in the first 24 hours post-CEA) ipsilateral stroke. After a mean follow-up time of 39.6 ± 16.4 months, 7.7% of patients (n = 17) had suffered one or multiple postoperative ipsilateral events (five TIAs, 12 strokes). Only one of these patients had a restenosis that needed treatment.

Patients who had an ipsilateral minor/major stroke during follow-up showed a lower plaque content of elastin (41.6 ± 17.5 mg/g *vs* 68.5 ± 54.5 mg/g, P .033), whereas no significant differences were observed for collagen and MMPs (Tables 3 and 4).

**Table 3:** Postoperative (>24 h after CEA) ipsilateral events in relation to collagen and elastin content (mean ± standard deviation) expressed in mg/g.

	Collagen	P	Elastin	P
Ipsilateral stroke				
*Yes (n = 12)*	52.5 ± 18.6	.807	41.6 ± 17.5	.033
*No (n = 209)*	54.6 ± 29.8		68.5 ± 54.5	
Ipsilateral TIA				
*Yes (n = 5)*	47.6 ± 20.7	.597	52.4 ± 41.8	.347
*No (n = 216)*	54.6 ± 29.5		67.3 ± 53.8	

**Table 4:** Postoperative (>24 h after CEA) ipsilateral events in relation to MMPs content (mean ± standard deviation) expressed in arbitrary units/g.

	MMP-1	P	MMP-3	P	MMP-9	P	MMP-12	P
Ipsilateral stroke								
*Yes (n = 12)*	211.83 ± 284.05	.821	20 ± 20.79	.595	1477.80 ± 2554.18	.683	28538.84 ± 24757.58	.945
*No (n = 197)*	226.61 ± 345.09		14.14 ± 16.01		921.36 ± 1247.18		25823.62 ± 20418.55	
Ipsilateral TIA								
*Yes (n = 5)*	144.53 ± 156.81	.653	7.91 ± 4.53	.549	352.47 ± 162.5	.491	19993.51 ± 5723.69	.903
*No (n = 204)*	227.75 ± 344.55		14.64 ± 16.47		968.04 ± 1364.9		28560.74 ± 24713.15	

**Fig 2:**
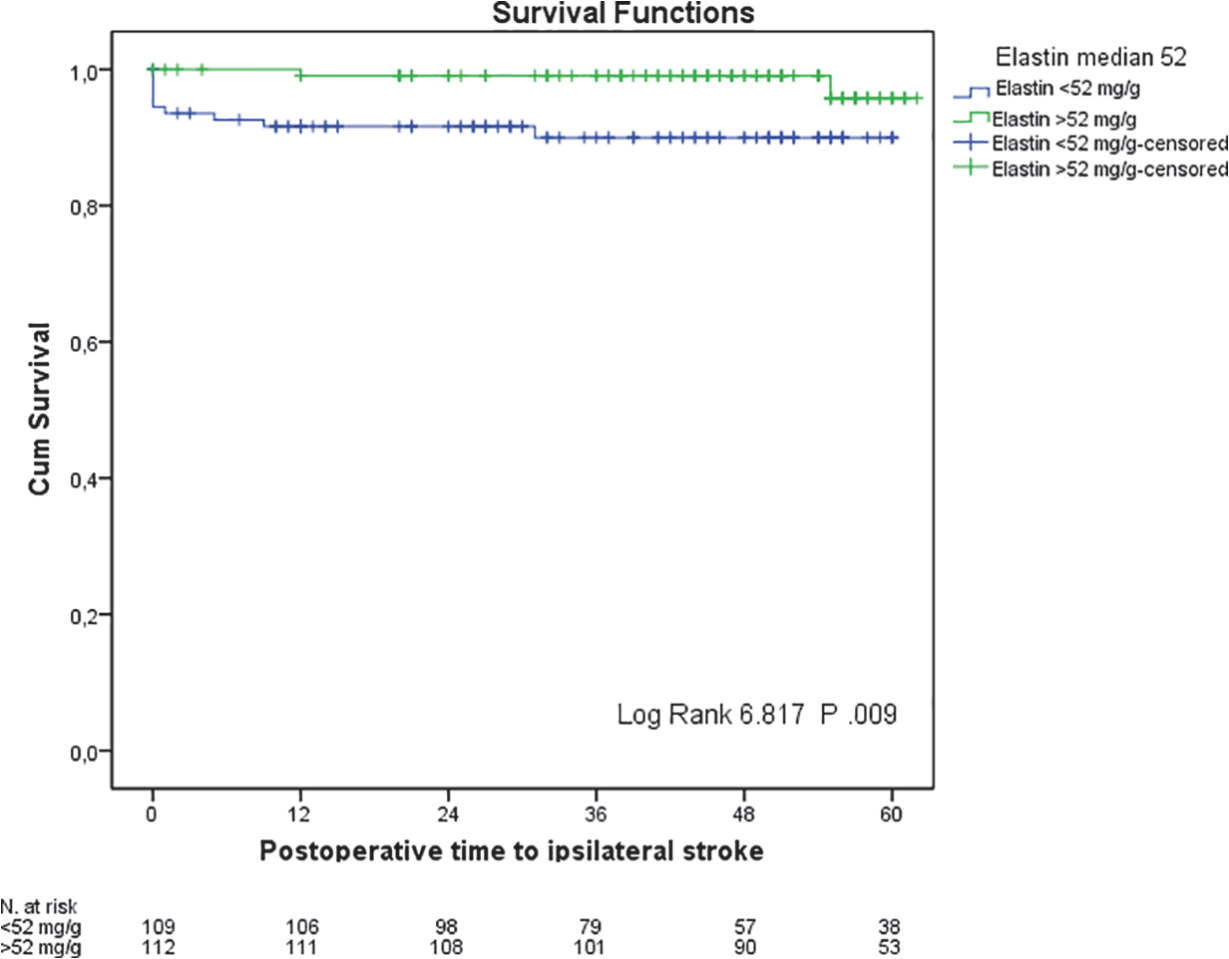
Kaplan-Meier survival analysis with median elastin content of 52 mg/g as a cut-off, and time to ipsilateral stroke.

Kaplan–Meier curves of event-free survival showed a significant increased incidence of ipsilateral stroke in patients with plaques that had an elastin content lower than median (52 mg/g, P 0.009 using Log Rank Chi-square test; Fig. 2). The higher risk for ipsilateral stroke remained significant when controlling for age, gender, hypertension, diabetes, smoking and pre-operative symptoms in a Cox Proportional Hazard model (hazard ratio 7.38, 95% C.I. 1.50–36.31).

When patients suffering an ipsilateral stroke in the first 72 hours after CEA were excluded from the analysis, the above-mentioned survival analysis findings were not confirmed.

## Discussion

The main finding of the present study is that a low carotid plaque content of elastin, as assessed by analyses of tissue removed at CEA, is associated with a higher risk of developing ipsilateral neurological symptoms, both pre- and postoperatively. Furthermore, in the current study population, the plaque content of elastin decreased with age and was lower in male and diabetic patients.

Even if CEA has been shown to be effective in preventing the occurrence of ipsilateral symptoms, this procedure does not eliminate the risk of neurological events. In the last decades the efforts of many researchers have focused on the risk stratification of patients with carotid artery stenosis, [13–14] but local atherosclerotic plaque characteristics have been poorly investigated in relation to future neurological events.

The majority of studies focusing on the composition of atherosclerotic plaques have been based on histology. As previously described, [10]. histological analysis has several advantages, allowing the identification and localization of specific proteins by immunohistochemistry. However, in our opinion, analysis of total levels of plaque components is more appropriately done by biochemical assays of whole plaque homogenates, since any issues of representativeness of the histology section are overcome. Furthermore, quantitative techniques such as ELISA are more reproducible and allow validation among different centers. [15]

When analyzing plaque composition, different confounding factors as temporal phenotypic alterations, should be taken into account. [16] The composition of the arterial wall results from a continuous interchange of cellular and non-cellular components. As previously reported, [17–18] the degradation or remodeling of the ECM components is mediated by matrix metalloproteinases (MMPs), a family of zinc-dependent endopeptidases, which play a key role in determining plaque instability. [19–20]. A wide spectrum of inflammatory cytokines and growth factors induce MMP transcriptional activation, [21–22] as confirmed by our findings of higher levels of MMP-1 and MMP-9 in plaques of patients who have suffered of neurological symptoms. Additionally, the elastic properties of the arterial wall, and in particular the content of elastin and collagen, may be also affected by various risk factors such as hypertension, diabetes mellitus, obesity, smoking, hypercholesterolemia, and kidney disease. [23] In this sense, we can explain our findings of a lower concentration of elastin, collagen and MMP-12 in diabetic patients who are known to have a higher incidence of pre- and postoperative events. [24] On the contrary, the higher levels of elastin, and collagen in women who also showed a lower incidence of postoperative events, could be explained through the known pro-elastic effect of female hormones on arterial wall properties. [25]

Moving the focus of our investigation to the possible influence of local atherosclerotic plaque characteristics on neurological outcome, we could not confirm our previous findings of high elastin content as a marker of plaque vulnerability. [11] On the contrary, elastin content was found to be lower in plaques that have produced neurological symptoms. This finding was indirectly confirmed through the detection of higher elastin content in plaques of patients who did not experience ipsilateral strokes either perioperatively or after a three-year follow-up period, even after correction for known risk factors. The difference in elastin levels when compared to our previous experience is most likely explained by differences in preparation of the homogenates which included additional centrifugation in the previous study. Another explanation of this difference can be the different extent of action of elastolytic proteases in the plaque of patients included in the current study. This hypothesis of the possible role of played by the interaction between these two components of the ECM was reinforced by the positive correlation between elastin and MMP-12 levels. However, the finding of their decreased levels in patients with preoperative symptoms is not in line with recent reports of overexpressed MMP-12 in atherosclerotic plaques associated with ischemic stroke. [26] The positive correlation between the levels of elastin and MMP-12 in plaques is partially unexpected since MMP-12 is a protease that degrades elastin. However, several factors may influence the contents of both elastin and MMP-12 and functions may not reflect absolute levels. Furthermore, this correlation could be the result of a possible feedback loop interaction of these two components of the ECM.

The significantly higher levels of collagen in patients who underwent CEA one or more months after the occurrence of neurological symptoms can be also interpreted as a sign of plaque remodeling. Furthermore, the expression of collagen could produce a stabilizing effect, limiting the occurrence of late neurological events as well as protecting against intraoperative events, which can result from surgical plaque manipulation. In the case of elastin, only a non-significant positive correlation to the time between symptoms and surgery could be detected. As previously stated, plaque composition is highly influenced by temporal phenotypic alterations, as it results from continuous remodeling processes engaged in the response to plaque rupture/healing. [27]

There are some limitations of the present study that should be considered. Most importantly, the study cohort was relatively small and the number of post-operative events only 17. The incoherency between the measurements of elastin and macrophage elastase (e.g. MMP-12) also has impact on the reliability of the present findings. It would be reasonable to assume that there would be an inverse association between MMP-12 and the plaque elastin content. Instead, we unexpectedly observed a positive correlation. However, it should be kept in mind that elastin turnover involves a number of other factor including expression of growth factors, MMP inhibitors as well as number and types of cells in the plaques. We have previously found a positive association between the plaque contents of platelet-derived growth factor and elastin suggesting that may have an important role. [28] Accordingly the findings need to be confirmed in larger studies with even longer follow-up and more in depth studies of the factors regulating plaque elastin expression are also warranted. Moreover, our measurements could have been influenced by the fact that we assessed elastin and collagen contents in homogenates of the entire plaque. It was therefore not possible to differentiate between possibly rupture-prone and stable areas. The combination of a histologic analysis of multiple plaque sections to quantify elastin and collagen content at different levels could have helped in the regional analysis of the content of these two proteins. However it would not be technically possible to perform simultaneous complete histologic and biochemical quantitative analyses on the same plaque.

The finding that patients with a low plaque content of elastin have an increased risk of developing an ipsilateral stroke during the follow-up could not be confirmed when excluding those five patients who suffered an ipsilateral stroke perioperatively, i.e. between 24 and 72 hours after CEA. However, this could suggest the instability of a plaque with a lower content of elastin, both in the short and the long term.

In conclusion, this study supports the concept that elastin seems to play a key role in plaque stabilization and destabilization. The need for closer follow-up and more aggressive secondary prevention of patients with reduced elastin content in harvested carotid plaques should be further investigated.

## References

[1] Clinical alert: benefit of carotid endarterectomy for patients with high-grade stenosis of the internal carotid artery. National Institute of Neurological Disorders and Stroke Stroke and Trauma Division. North American Symptomatic Carotid Endarterectomy Trial (NASCET) investigators. Stroke; a journal of cerebral circulation. 1991;22(6):816–7. 2057984 10.1161/01.str.22.6.816

[2] Endarterectomy for asymptomatic carotid artery stenosis. Executive Committee for the Asymptomatic Carotid Atherosclerosis Study. JAMA: the journal of the American Medical Association. 1995;273(18):1421–8. 7723155

[3] Randomised trial of endarterectomy for recently symptomatic carotid stenosis: final results of the MRC European Carotid Surgery Trial (ECST). Lancet. 1998;351(9113):1379–87. 9593407

[4] Greenwald SE. Ageing of the conduit arteries. The Journal of Pathology. 2007;211(2):157–72. 17200940 10.1002/path.2101

[5] McEniery CM, Wilkinson IB, Avolio AP. Age, hypertension and arterial function. Clinical and experimental pharmacology & physiology. 2007;34(7):665–71. 17581227 10.1111/j.1440-1681.2007.04657.x

[6] Payne RA, Wilkinson IB, Webb DJ. Arterial stiffness and hypertension: emerging concepts. Hypertension. 2010;55(1):9–14. 10.1161/HYPERTENSIONAHA.107.090464 19948990

[7] Hansen F, Bergqvist D, Lindblad B, Lindh M, Matzsch T, Lanne T. Accuracy of duplex sonography before carotid endarterectomy—a comparison with angiography. European journal of vascular and endovascular surgery: the official journal of the European Society for Vascular Surgery. 1996;12(3):331–6. 10.1016/s1078-5884(96)80252-8 8896476

[8] Asciutto G, Wistrand J, Riva L, Bjorses K, Goncalves I, Dias NV. Long-term progression of contralateral carotid artery disease after endarterectomy: is there a need for Duplex surveillance? International angiology: a journal of the International Union of Angiology. 2012;31(4):361–7. 22801402

[9] Ludvigsson JF, Andersson E, Ekbom A, Feychting M, Kim JL, Reuterwall C, et al External review and validation of the Swedish national inpatient register. BMC public health. 2011;11:450 10.1186/1471-2458-11-450 21658213 PMC3142234

[10] Goncalves I, Lindholm MW, Pedro LM, Dias N, Fernandes e Fernandes J, Fredrikson GN, et al Elastin and calcium rather than collagen or lipid content are associated with echogenicity of human carotid plaques. Stroke; a journal of cerebral circulation. 2004;35(12):2795–800. 15514195 10.1161/01.STR.0000147038.12073.59

[11] Goncalves I, Moses J, Dias N, Pedro LM, Fernandes e Fernandes J, Nilsson J, et al Changes related to age and cerebrovascular symptoms in the extracellular matrix of human carotid plaques. Stroke; a journal of cerebral circulation. 2003;34(3):616–22. 12624281 10.1161/01.STR.0000058157.69113.F6

[12] Rattik S, Wigren M, Björkbacka H, Fredrikson GN, Hedblad B, Siegbahn A, et al. High plasma levels of heparin-binding epidermal growth factor are associated with a more stable plaque phenotype and reduced incidence of coronary events. Arterioscler Thromb Vasc Biol. 2014 Oct 30. pii: ATVBAHA.114.304369. 10.1161/ATVBAHA.114.304369 25359857

[13] de Borst GJ, Vos JA, Reichmann B, Hellings WE, de Vries JP, Suttorp MJ, et al The fate of the external carotid artery after carotid artery stenting. A follow-up study with duplex ultrasonography. Eur J Vasc Endovasc Surg. 2007;33(6):657–63. 17337347 10.1016/j.ejvs.2007.01.010

[14] Hellings WE, Pasterkamp G, Verhoeven BA, De Kleijn DP, De Vries JP, Seldenrijk KA, et al Gender-associated differences in plaque phenotype of patients undergoing carotid endarterectomy. J Vasc Surg. 2007;45(2):289–96; discussion 96–7. 17264005 10.1016/j.jvs.2006.09.051

[15] Hellings WE, Pasterkamp G, Vollebregt A, Seldenrijk CA, De Vries JP, Velema E, et al Intraobserver and interobserver variability and spatial differences in histologic examination of carotid endarterectomy specimens. J Vasc Surg. 2007;46(6):1147–54. 17950565 10.1016/j.jvs.2007.08.018

[16] Niciforovic-Surkovic O, Ac-Nikolic E, Ukropina S, Mijatovic-Jovanovic V. [Physical activity of school children and their parents in Vojvodina]. Medicinski pregled. 2005;58(1–2):52–6. 18257206 10.2298/mpns0502052n

[17] Grujic V, Martinov-Cvejin M, Ac-Nikolic E, Niciforovic-Surkovic O. [Epidemiology of obesity in the adult population of Vojvodina]. Medicinski pregled. 2005;58(5–6):292–5. 16526237 10.2298/mpns0506292g

[18] Sala-Newby GB, George SJ, Bond M, Dhoot GK, Newby AC. Regulation of vascular smooth muscle cell proliferation, migration and death by heparan sulfate 6-O-endosulfatase1. FEBS Lett. 2005;579(28):6493–8. 16289059 10.1016/j.febslet.2005.10.026

[19] Newby AC, Johnson JL. Genetic strategies to elucidate the roles of matrix metalloproteinases in atherosclerotic plaque growth and stability. Circ Res. 2005;97(10):958–60. 16284186 10.1161/01.RES.0000193565.23357.c0

[20] Newby AC. Matrix metalloproteinases regulate migration, proliferation, and death of vascular smooth muscle cells by degrading matrix and non-matrix substrates. Cardiovascular research. 2006;69(3):614–24. 16266693 10.1016/j.cardiores.2005.08.002

[21] Johnson JL, George SJ, Newby AC, Jackson CL. Divergent effects of matrix metalloproteinases 3, 7, 9, and 12 on atherosclerotic plaque stability in mouse brachiocephalic arteries. Proc Natl Acad Sci U S A. 2005;102(43):15575–80. 16221765 10.1073/pnas.0506201102 PMC1266110

[22] Bond M, Sala-Newby GB, Wu YJ, Newby AC. Biphasic effect of p21Cip1 on smooth muscle cell proliferation: role of PI 3-kinase and Skp2-mediated degradation. Cardiovasc Res. 2006;69(1):198–206. 16212951 10.1016/j.cardiores.2005.08.020

[23] Newby AC. Studying mechanisms underlying shedding of endothelial membrane proteins could help patients at risk for myocardial infarction. Cardiovasc Res. 2005;67(1):4–5. 15907817 10.1016/j.cardiores.2005.04.024

[24] Johnson TW, Wu YX, Herdeg C, Baumbach A, Newby AC, Karsch KR, et al Stent-based delivery of tissue inhibitor of metalloproteinase-3 adenovirus inhibits neointimal formation in porcine coronary arteries. Arterioscler Thromb Vasc Biol. 2005;25(4):754–9. 15681295 10.1161/01.ATV.0000157582.33180.a9

[25] Vitale C, Mendelsohn ME, Rosano GM. Gender differences in the cardiovascular effect of sex hormones. Nature reviews Cardiology. 2009;6(8):532–42. 10.1038/nrcardio.2009.105 19564884

[26] Traylor M, Mäkelä KM, Kilarski LL, Holliday EG, Devan WJ, Nalls MA, et al A novel MMP12 locus is associated with large artery atherosclerotic stroke using a genome-wide age-at-onset informed approach. PLoS Genet. 2014 Jul 31;10(7):e1004469 10.1371/journal.pgen.1004469 25078452 PMC4117446

[27] Peeters W, Hellings WE, de Kleijn DP, de Vries JP, Moll FL, Vink A, et al Carotid atherosclerotic plaques stabilize after stroke: insights into the natural process of atherosclerotic plaque stabilization. Arterioscler Thromb Vasc Biol. 2009 Jan;29(1):128–33. 10.1161/ATVBAHA.108.173658 18931283

[28] Edsfeldt A, Goncalves I, Grufman H, Nitulescu M, Duner P, Bengtsson E, et al Impaired fibrous repair: a possible contributor to atherosclerotic plaque vulnerability in patients with type II diabetes. Arterioscler Thromb Vasc Biol 2014 Sept;34(9):2143–50. 10.1161/ATVBAHA.114.303414 25035341

